# Recent Fracture of Skull with Depression

**Published:** 1883-06

**Authors:** 


### Recent Fracture of Skull with Depression.

Dr. S. W. Gross (*Am. Practitioner*): In all recent fractures of the skull with depression, if the latter be moderate, whether simple or compound, the patient should be left alone. If, however, fixed and severe pain at the point of injury, febrile excitement, increase of local temperature and a commencing puffiness of the scalp, supervene within a few days after the accident—signs which are indicative of depression of the internal table and the development of pathymeningitis—elevation of the depression should be promptly effected. In all recent fractures, whether simple or compound, attended with symptoms of compression, the trephine should be resorted to, and the same rule should apply, whether symptoms be present or not, if the depression be considerable and funnel-shaped.

